# Crack Detection in Concrete Tunnels Using a Gabor Filter Invariant to Rotation

**DOI:** 10.3390/s17071670

**Published:** 2017-07-20

**Authors:** Roberto Medina, José Llamas, Jaime Gómez-García-Bermejo, Eduardo Zalama, Miguel José Segarra

**Affiliations:** 1 CARTIF Foundation, Parque Tecnológico de Boecillo, 47151 Valladolid, Spain; robmed@cartif.es (R.M.); joslla@cartif.es (J.L.); 2 ITAP-DISA, University of Valladolid, 47002 Valladolid, Spain; ezalama@eii.uva.es; 3 DRAGADOS S.A., Av. Camino de Santiago 50, 28050 Madrid, Spain; mjsegarra@dragados.com

**Keywords:** tunnel inspection, concrete surface, image processing, spatial frequency domain

## Abstract

In this article, a system for the detection of cracks in concrete tunnel surfaces, based on image sensors, is presented. Both data acquisition and processing are covered. Linear cameras and proper lighting are used for data acquisition. The required resolution of the camera sensors and the number of cameras is discussed in terms of the crack size and the tunnel type. Data processing is done by applying a new method called Gabor filter invariant to rotation, allowing the detection of cracks in any direction. The parameter values of this filter are set by using a modified genetic algorithm based on the Differential Evolution optimization method. The detection of the pixels belonging to cracks is obtained to a balanced accuracy of 95.27%, thus improving the results of previous approaches.

## 1. Introduction

Traditional methods of tunnel inspection are based on the subjective criteria of a human operator that performs local visual inspection and sensing equipment that usually requires installation and contact with the tunnel surface (plaster indicators, crackmeters, etc.). The visual inspection of 100% of the tunnel surface would be preferred, but this requires the management of a massive amount of information. Therefore, the automatic acquisition and processing of images emerges as the only practical solution. To this end, the development of image processing algorithms capable of detecting the presence of defects in the surface of the tunnel is necessary.

In this paper, the automated acquisition and processing of images of 100% of the surface of the tunnel is proposed that improves upon the results obtained with traditional techniques. The management of a large amount of information provided by the image sensors on the entire surface of the tunnel does not allow the manual evaluation of data, so new methods for the automated processing of this data have been developed. The proposed methods allow for the inspecting and detecting of fissures and cracks in the surface of the tunnel. This type of defect is one of the most usual, but also one of the most difficult to detect.

The image sensors are mounted onto an inspection platform designed to inspect any rail, highway, or hydraulic tunnel whose surface is made from concrete by tunnel boring machines. The methodology for the inspection of different tunnel typologies and different crack width detection requirements is addressed. A new method based on Gabor filters for the detection of cracks, independently of their direction of growth, is also presented.

## 2. Related Work

A number of techniques have focused on the detection of defaults in continuous surfaces by using computer vision-based sensors. Some of these techniques are based on the segmentation of the original image, either by using a fixed threshold as in [1], a threshold computed from the previous images as in [2,3], or a different threshold for each image column, as in [4]. Other authors have worked on the previously processed images, as in [2,5,6]; some have worked on entropy [7], where the use of the Hough transform is proposed; or on morphological filters, as in [8]; or on different edge detection filters, as in [9,10,11].

More specifically, the detection of cracks has been addressed in many areas, such as the inspection of concrete and asphalt infrastructures. One of the most developed fields is the detection of cracks in roads, these being one of the infrastructures with the largest surface anywhere in the world. The simplest and most common detection method is direct image segmentation, given that cracks are usually darker than the surrounding areas. Morphological filters are then applied to the segmented images in order to join the cracks and remove the segmentation noise, as is done for example in [1]. Other works have addressed a more elaborate segmentation, such as [12], where the Neighboring Difference Histogram Method (NDHM), which compares each pixel with the histogram of the surrounding pixels, is used; or [6], where the images are divided into different zones, to which a modified Otsu thresholding algorithm is applied [13], and the resulting entropy is computed. Other methods are based on the search for seeds that are merged by taking into account the fact that the cracks appear as elongated structures [14]. Recently, Convolutional Neuronal Networks has been used to detect cracks, as can be found in [15], where cracks are detected at pixel level.

A significant number of works can also be found where the detection of cracks in concrete buildings and infrastructures, especially bridges, is addressed. Four methods for the detection of cracks in concrete bridges are compared in [16]: Sobel and Canny edge detectors, the Fourier transform, and the Haar wavelet transform. These authors conclude that the Haar wavelet transform is the best performer.

A method for detecting cracks in concrete structures is described in [17]. This method is based on the application of a large number of filters to a database that has previously been processed by hand. The optimal combination of filters to obtain the best possible result is selected using genetic algorithms. In [10], a method for detecting cracks in concrete surfaces by areascan color sensors, based on different edge detection algorithms, is described. The system can detect cracks with an opening greater than 0.25 mm.

The works where cracks are detected inside tunnels differ from the others mainly due to the use of artificial light. Some works are based on thresholding techniques, such as [4], where the value of each pixel is compared to the average value of the pixels in the column where that pixel is located. The work of Fujita et al. [11], cited above, can be included in this group. The main drawback of these techniques is how to choose the threshold value. The methods for setting the threshold value are usually based on prior knowledge, but these methods are not generalizable and do not usually work properly when applied to real tunnels, where shadows and humidity may occur.

Other techniques for detecting cracks in tunnels are based on models, such as the one described above in [3], using the concept of percolation, or the one presented in [18], where it is assumed that cracks are formed by line segments joined together. In the second case, the image resolution is 2.5 mm per pixel and can be considered a semi-automatic method, as it needs human intervention to choose the seeds of every crack.

There are also some methods based on pattern recognition, such as the one proposed in [19], where the SVM (Support Vector Machine) algorithm is used to determine whether the preselected areas are really cracks. An inspection system of concrete tunnels composed of nine linear color CMOS cameras, whose images are transformed to gray images for processing, is proposed in [8]. First, a smoothing filter to remove noise is applied to the gray images. The resulting image is filtered by the top-hat filter to enhance cracks, as they are supposed to be darker than the rest of the image. The threshold for segmenting the filtered image is set by analyzing the distribution of the gray levels of the filtered image. Neighboring pixels are grouped, and shape and gray level features are calculated to determine which groups are really cracks and which are not. For this, four pattern recognition techniques are applied: two neural networks, the SVM algorithm, and the nearest neighbor algorithm. The accuracy of these algorithms is approximately 90%, which was calculated using 200 pixel groups, of which 64 were actually cracks. A total of nine linear cameras of 12,288 pixels acquire images in a subway tunnel covering 270° of the tunnel, but the tunnel diameter was not available, so the image resolution cannot be determined.

Table 1 summarizes the described related work. However, in general, the reviewed techniques are often not suitable for the detection of cracks in tunnels. The roughness of the concrete, the shadows derived from this roughness, and the fact that the crack brightness is often similar to that of the surrounding areas mean that these segmentation techniques can hardly provide the required results.

In the present paper, a segmentation process based on the localized space-frequency information of the images, obtained using Gabor filters, is presented. Gabor filters have also been used successfully by our research team for the detection of cracks in roads [20]. However, in this previous work, the sensed images are divided into pieces and the algorithm determines whether there is a crack inside each piece using a variant of the AdaBoost algorithm for training. The present work addresses the use of Gabor filters in a significantly different way. In particular, the classification process is made for every pixel of the image, and the Gabor filter parameters are set using a modified genetic algorithm based on the Differential Evolution optimization method. Moreover, the texture of asphalt roads and concrete surfaces are very different, and while road cracks exhibit longitudinal and transverse preferential directions, tunnel cracks can be oriented in any direction. Therefore, a new method based on Gabor filters, which we have called Gabor filter invariant to rotation, is proposed in the present work for the detection of cracks independent of their direction of growth. 

## 3. System Description

There are multiple defects that may appear on the surface of the lining of a concrete tunnel. These defects can be classified into four main types: mechanical, chemical, water-related and joint-related defects.

**Figure 1:**
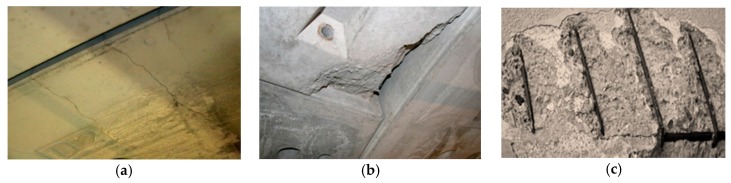
Mechanical defects in tunnels: (**a**) fissures; (**b**) detachments on precasts; (**c**) seen steel frames.

Concerning the mechanical defect type, there are fissures that appear in the surface mainly due to the presence of tensions over its resistance capacity. When a fissure traverses the entire element thickness, from side to side, it becomes a crack. Other mechanical defects are detachments or ruptures on precasts, broken off corners, or seen steel frames. Some examples of these defects are shown in Figure 1. Finally, other defects are of a chemical nature, such as carbonation, efflorescence, and other defects related to the presence of water, such as moisture, dripping, and seepage.

This paper aims to develop a system for the detection of fissures and cracks in tunnels. This type of defect can be identified through visual inspection, but often goes unnoticed during a routine human inspection. In addition, these defects appear at preliminary phases of tunnel deterioration, so their early detection allows preventive actions to be taken, before the problem becomes more severe. Moreover, both fissures and cracks exhibit a similar visual appearance. Therefore, from now on, we will use the term *crack* for referring to both fissures and cracks. 

Cracks can be classified into different categories depending on their opening and the apparent damage of the precast, as shown in Table 2. Cracks of type 0 are those with an opening of less than 0.1 mm. Cracks of type A include those with an opening of between 0.1–0.4 mm, that do not reach the intrados steel, and so are of an aesthetic nature. Type B cracks have an opening smaller than 2 mm at their head, pass through the first reinforcement layer, and are stabilized. Finally, type C cracks correspond to the multicracked precasts. Subtype C1 corresponds to cracks without risk of fall or detachment, usually with the presence of moisture or water ooze, and subtype C2 correspond to multicracked precasts with risk of detachment.

**Figure 2:**
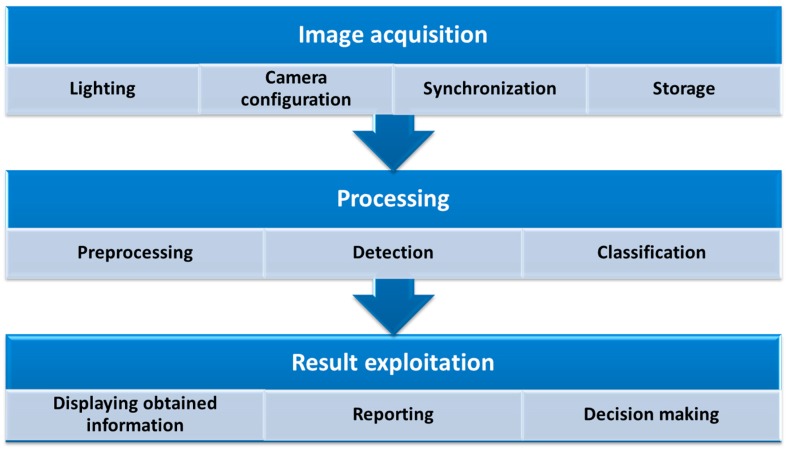
Stages of the automatic visual inspection process.

A system for the automatic visual inspection of tunnels consists of three major blocks or stages: image acquisition, processing, and result exploitation. Figure 2 shows these major blocks and the main actions involved in each of them.

Image acquisition is addressed in depth in Section 4 of this paper, while the processing techniques for crack detection in concrete tunnels are described in Section 5. The obtained results can be visualized, and reports of the state of the tunnel can be made, allowing engineers to take the pertinent action to repair them.

## 4. Image Sensors for Crack Detection

Concerning data acquisition, the use of linear cameras is recommended in order to simplify lighting and ulterior data processing. These cameras are equipped with a 1D sensor array (instead of the 2D array used in common cameras). The linear cameras must be attached to a mobile platform that travels along the tunnel. The number of cameras, their resolution, and their arrangement will depend on the size and characteristics of the tunnel and on the minimum size of the cracks to be detected.

In particular, in order to achieve a uniform resolution along the entire perimeter of the tunnel, the cameras must be arranged radially, i.e., placed on a circumference centered at the tunnel center (for the current tunnel section), and oriented so that their optical axes cross at the said center. Moreover, the inspection elements must be placed in the area of the tunnel free from obstacles, which we will call the Working Area (WA). The radius of the bigger circumference inside this area is the working area radius, *R_WA_*. 

The camera sensor type and the corresponding optics must be selected according to the minimum crack size, as will be explained later. These aspects will determine the working distance, *W_D_*, namely the distance between the camera’s optical center and the tunnel surface. In general, this working distance is recommended to be lower than the radius of the tunnel, *R_T_*, plus the radius of the working area, *R_WA_*, and greater than the radius of the tunnel, *R_T_*, minus the radius of the working area, *R_WA_*:

(1) $$R_{T}-R_{WA}<W_{D}<R_{T}+R_{WA}$$

**Figure 3:**
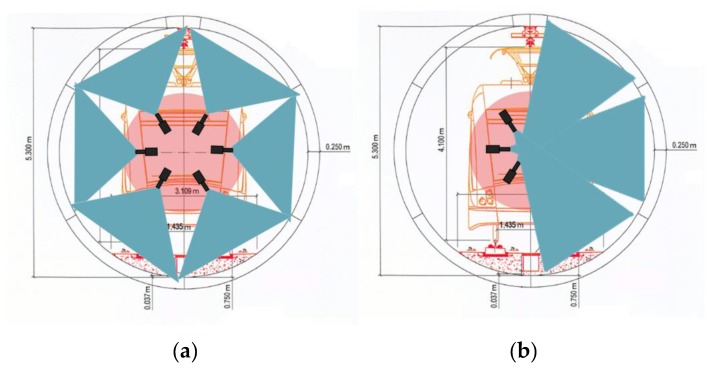
Two examples of camera position distribution, and the portion of the tunnel section covered by each camera. (The field-of-view of the cameras is shadowed in blue). (**a**) Working distance (*W_D_*) is smaller than the radius of the tunnel (*R_T_*) (**b**) Working distance (*W_D_*) is greater than the radius of the tunnel (*R_T_*), so only half of the cameras can be placed in the same plane.

When the selected working distance is greater than the tunnel radius, the cameras should be placed along half the tunnel section so that their view is not obstructed by the opposite cameras, as described in Figure 3. Moreover, either the system should travel twice along the tunnel or the cameras should be arranged on two different planes.

Concerning camera sensor resolution, common linear cameras range from about 1024 pixels for an e2v AviiVA SM2 1010, for example, to 12,288 pixels for an e2v AviiVA UM8 camera. The number of cameras required for inspection is determined by the actual sensor resolution and the minimum crack size:

(2) $$N_{C}>\frac{2P_{T}}{W_{C}^{\min}N_{P}}$$

In this expression, *N_C_* is the number of cameras, *P_T_* is the tunnel perimeter, *W_C_*^min^ is the minimum crack width, and *N_P_* is the number of pixels of the camera sensor. Moreover, a (small) overlap between the field of view of two adjacent cameras is recommended to avoid gaps. 

The necessary sensor resolution that allows the camera to measure these types of crack requires twice the resolution of the image, i.e., to detect cracks 1 mm wide, images with a resolution of at least 2 pixels per millimeter should be acquired. Typical minimum crack values are between 0.1 and 2 mm, which would include most types of cracking defects and cracks in tunnels. 

Furthermore, appropriate optics must be selected for the chosen camera, so the image can be captured within the established working distance limits, and the tunnel surface can be properly focused. 

Table 3 reports some examples of sensor camera resolution, number of cameras and focal length of the optics recommended for detecting cracks ranging from 0.1 to 2 mm, which cover most expected crack types, for three tunnel types: electricity tunnel, underground tunnel and high-speed train tunnel (sized 3.5 m, 5.3 m, and 10.40 m in diameter, respectively). Both one-pass and two-pass acquisition cases are shown.

Finally, the data captured by the cameras should be stored for later analysis. In the worst-case scenario, for the 10.4 m sized, high-speed train tunnel, with 0.1 mm cracks, about 8 TBytes are required to store images from a 1 km tunnel. These storage requirements drop to 0.5 Tbytes in the case of 0.4 mm cracks. The storage requirements drop significantly for the other resolution figures.

## 5. Methodology for Spatial-Frequency Image Analysis by Gabor Filters Invariant to Rotation

### 5.1. Gabor Filter

A two-dimensional Gabor filter is a complex sinusoidal wave modulated by a Gaussian envelope [21]. The filter performs a localized and oriented frequency analysis of a two-dimensional signal. The spatial domain formulation is,

(3) $$\begin{array}{l} G_{\sigma ,F,\vartheta }(x,y)=g_{\sigma }(x,y)\exp\left[j2\pi Fx^{\prime }\right]\\ \mathrm{where}\;g_{\sigma }(x,y)=\frac{1}{2\pi \sigma_{x}\sigma_{y}}\exp\left[-\frac{1}{2}\left({\left(\frac{x^{\prime }}{\sigma_{x}}\right)}^{2}+{\left(\frac{y^{\prime }}{\sigma_{y}}\right)}^{2}\right)\right]\\ \mathrm{and}\;x^{\prime }=x\cos\theta +y\sin\theta \;y^{\prime }=-x\sin\theta +y\cos\theta \end{array}$$

where *x*, *y* are the spatial coordinates, *F* is the central frequency, *θ* is the angle between the direction of the sinusoidal wave and the *x*-axis, and *σ_x_* and *σ_y_* are the smoothing parameters (standard deviations of the Gaussian envelope in the direction of the wave and orthogonal to it). The frequency domain formulation of the Gabor filter is,

(4) $$\begin{array}{l} G_{\sigma ,F,\theta }(u,v)=\exp\left[\frac{-1}{2}\left(\frac{{\left(u^{\prime }-F\right)}^{2}}{\sigma_{u}^{2}}+\frac{{v^{\prime }}^{2}}{\sigma_{v}^{2}}\right)\right]\\ \mathrm{where}\;\sigma_{u}=\frac{1}{2\pi \sigma_{x}}\;,\;\sigma_{v}=\frac{1}{2\pi \sigma_{y}}\\ \;u^{\prime }=u\cos\theta +v\sin\theta \;\mathrm{and}\;v^{\prime }=-u\sin\theta +v\cos\theta \end{array}$$

Four parameters must be set to appropriate values in order to apply a Gabor filter for processing an image: the center frequency, *F*, the orientation, *θ*, and two smoothing parameters, *σ_x_* and *σ_y_*. We actually aim to enhance the cracks in images, so the filtered image should offer high values in the presence of cracks and low values otherwise. This can be achieved by selecting the frequency value so the corresponding wavelength (*2π/F*) approaches the width of the crack, and an orientation value so it matches that of the crack. In turn, smoothing parameters serve to increase or decrease the range of frequencies and orientations to which the filter reacts.

### 5.2. Gabor Filters Invariant to Rotation

Tunnel cracks can exhibit any possible orientation in the image, and a single crack can even exhibit different orientations along its length. Therefore, the design of filters invariant to rotation (i.e., filters that have the same response regardless of the orientation of the cracks) has been addressed in the present work. To do this, a given number of different orientations is assumed, *n_θ_*, and a set of values for *F*, *σ_x_*, and *σ_y_* parameters is selected. Then, a filtered image is obtained for each orientation in *n_θ__¡_* and the remaining parameter values. The resulting filtered-images are combined to obtain a final, single image by taking the maximum value at each pixel,

(5) $$I_{ij}^{F}=Max\left(I_{ij}^{n}\right)$$

where *I^F^* is the final filtered image, *I^n^* is the image filtered by the Gabor filter with the *n^th^* orientation, and *i* and *j* are the columns and rows of the image, respectively. Finally, this resulting image, *I^F^*, is segmented by computing a suitable threshold value.

**Figure 4:**
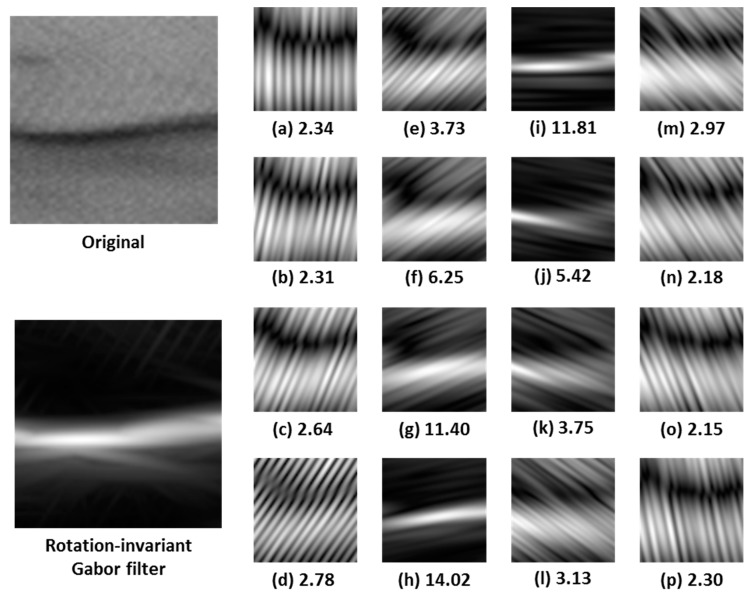
Example of the application of the rotation-invariant Gabor filter. The original image and the result of applying the rotation-invariant Gabor filter to it are shown on the left. The Gabor filters applied along 16 orientations (from 0° to 180°) are shown on the right, along with their maximum values (**a**–**p**).

An example of the application of the rotation-invariant Gabor filter is shown in Figure 4. The original image and the result of applying the rotation-invariant Gabor filter to it are shown on the left. The Gabor filters applied along 16 orientations (from 0° to 180°) are shown on the right, along with their maximum values (images have been normalized for a better visualization). In this example, *F* = 0.16 pixels^−1^, *σ_x_* = 2.08 pixels and *σ_y_* = 17.16 pixels (see Section 6 for further details on these values).

### 5.3. Selection of Optimal Parameters by Differential Evolution Algorithm

A proper choice of the *F*, *σ_x_*, and *σ_y_* parameter values of the Gabor filters is essential to obtain accurate results. In the present work, the use of a genetic algorithm based on the so-called Differential Evolution optimization method is proposed. This algorithm was first introduced by Storn and Price [22], and allows good convergence results to be achieved, to a large extent avoiding falls in local minimums. The algorithm operates in four main steps:

1. **Initialization**: Generate *N_p_* vectors randomly within a given parameter space. Each vector is composed of the variables of the function to be minimized, i.e., the parameters of the image segmentation method whose optimization is looked for. The segmentation method is applied to a set of training images using each of these vectors, and the corresponding result is evaluated.
2. **Mutation**: *N_p_* new vectors are created by applying,
  (6) $$v_{i,g}=x_{best,g}+F(x_{r1,g}-x_{r2,g})$$
  where *i* is the vector number, *g* the generation number, *x* the current vector, and *v* the muted one. Moreover, the subscript *best* refers to the vector that leads to the best results, *r1* and *r2* are two different values randomly selected within the 0 to *N_p_*−1 range, and *F* is a mutation scale vector. The original algorithm proposed by Storn and Price used another random vector, *r3*, instead of the one leading to the best result, *best*, and *r1*, *r2*, *r3*, and *i* should be different.
3. **Recombination**: In this step, the mutated vector is recombined with the unmutated one by applying,
  (7) $$u_{ji,g}=\{\begin{array}{cc} v_{ji,g} & {\mathrm{if}\;\mathrm{rand}}_{j}\left[0,1\right]\leq CR\\ x_{ji,g} & \mathrm{otherwise} \end{array}$$
  where *j* is the variable number, *u* is the recombined vector and *CR* is the probability for a variable to mutate. It must be ensured that at least one variable is mutated, so one variable is initially mutated and the probability is applied to the others.
4. **Selection**: Finally, the recombined vectors are evaluated and the best performing one, from the original and recombined vectors, are assigned to the next generation as follows:
  (8) $$x_{i,g+1}=\{\begin{array}{cc} u_{i,g} & \mathrm{if}\;f(u_{i,g})<f(x_{i,g})\\ x_{i,g} & \mathrm{otherwise} \end{array}$$

In this expression, *f* is the function selected to evaluate the vector performance. The whole process is then repeated from point 2 until a stop criterion is matched.

The results of the algorithm are improved in the present work by modifying the initialization step of the above-described process (step 1) in such a way that a Gabor filter bank is used, computed as described in [20], instead of starting from a group of randomly selected vectors. This reduces the possibility of falls in local minimums and reduces the number of generations required to reach the stop criterion.

## 6. Experimental Results

### 6.1. Inspection Platform Prototype

**Figure 5:**
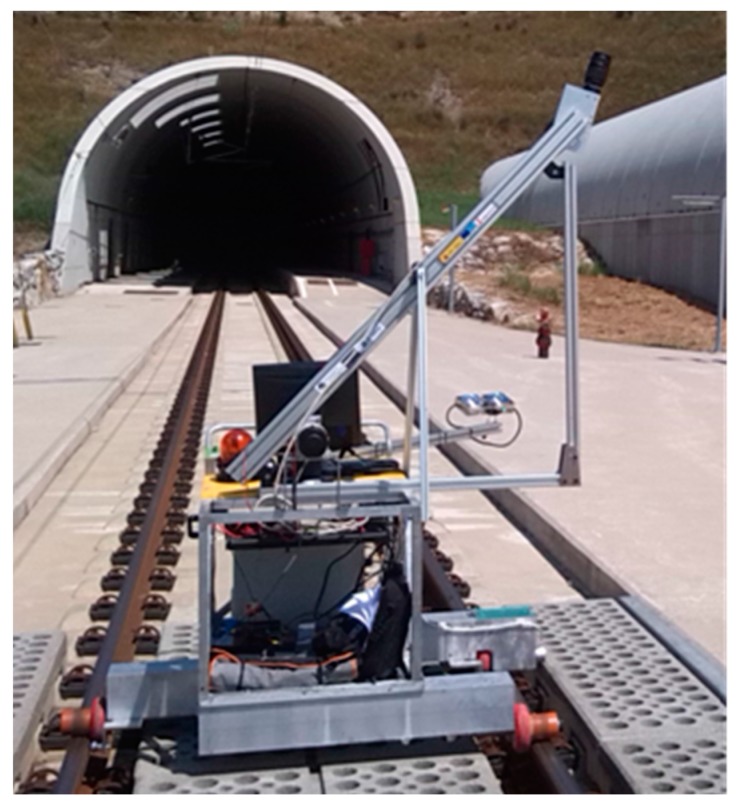
Prototype of the tunnel inspection platform.

A mobile platform that travels along the railroad track (see Figure 5) has been developed to test the feasibility of the proposed method. The platform is equipped with an Aviiva SM2 CL 1010 linear camera. Moreover, a Tamron AF 70–300 mm F/4–5.6 Di LD Macro 1:2 optics has been used, along with a 100 W halogen bulb with an AR111 reflector. The selected linear camera has a sensor resolution of 1024 pixels and can acquire up to 54,400 lines per second. The selected optics has a variable zoom that can be set from 70 to 300 mm, so it can be used in tunnels of different diameters. The reflector lighting system allows the light to be concentrated in an 8° cone. Both the camera and the light source can be oriented in multiple positions, which allows different areas of the tunnel to be acquired with different resolutions. The images of the tunnel surface obtained by using this system are then processed through the crack detection algorithms.

The camera is configured in such a way that the average value of defect-free images is placed at roughly half the dynamic range of the camera, i.e., 128 in a 0 to 255 range. Thus, defects both darker and brighter than the background surface are correctly observed. This set up can be achieved by properly tuning three configurable parameters of the camera: exposure time, aperture, and sensitivity. These parameters, which determine the intensity level of pixels in the image, also affect other factors such as image sharpness or noise level.

Moreover, the image acquisition must be synchronized with the movement of the platform in order to correctly compose the image. An incremental encoder is then required on the platform wheels to measure the platform displacement. The encoder signal is activated once all the cameras on board the platform are ready to acquire images. Thus, the first encoder pulse received by every camera is the same pulse for all of them, so proper synchronization is achieved.

A software module has been developed in the present work to ease the system setup and image grabbing. This module provides the status of the different cameras, the current vehicle speed and the maximum admissible speed the vehicle can travel, which is calculated as follows:

(9) $$S_{MAX}=\frac{R_{A}}{T_{Exp}}=\frac{W_{C}^{\min}}{2T_{Exp}}$$

where *S_MAX_* is the maximum admissible speed of the vehicle, *R_A_* is the resolution in the direction of movement of the vehicle, *T_Exp_* is the exposure time, and *W_C_*^min^ is the minimum crack width. A correction factor of 0.9 is then applied to the result in order to ensure that the maximum speed is never exceeded. For example, if exposure time is set to 100 µs and *W_C_*^min^ is 0.2 mm, it results in *S_MAX_* being 1 m/s. Appling the correction factor means that the vehicle speed should never be greater than 0.9 m/s.

In addition, another software module has been developed that provides all the required information concerning the acquired images and the processed data. The human supervisor can navigate the stored information using this software and analyze the result of the tunnel crack detection.

### 6.2. Crack Detection Results

The proposed crack detection algorithm has been trained and tested using a number of images containing cracks, extracted from a 3k rail tunnel survey. The pixels of these images have been manually classified as defect-free or cracks. A total of 100,000 pixels have been used, 20,000 of which are pixels with cracks. These pixels have been used to set the parameters of the proposed algorithms and verify the accurate operation of the system.

The parameters of the Gabor filter invariant to rotation have been set using the variant of the Differential Evolution algorithm described in the previous section. The following conditions have been assumed:

1. The first generation has been generated by defining a bank of filters covering the entire frequency space (instead of just taking a random selection). The parameters of the filter bank are: filter size (*N × M*) = 128 × 128 pixels, number of frequencies (*n_F_*) = 12, number of orientations (*n_θ_*) = 16, frequency band (*B_F_*) = 0.5 octaves, overlapping constant along *x (K_x_) =* 1, overlapping constant along *y* (*K_y_*) = 1, Max. Center Frequency (*F_M_*) = 0.427 pixels^−1^.
2. There are 12 individuals in each generation, *N_p_* = 12. This value matches the number of frequencies of the filter bank described in the previous section.
3. Mutation probability has been established at 50%, *CR* = 0.5.
4. The mutation scale factor *F* is not a fixed value, but a function of the current generation and the number of generations without improvement, according to,
  (10) $$F=1-\frac{n}{n_{max}}+\frac{n^{wi}}{n_{max}^{wi}}$$
  where *n* is the current generation, *n_max_* is the maximum number of generations, *n^wi^* is the number of generations without improvement, and *n^wi^_max_* is the maximum number of generations without improvement. Thus, the scale factor decreases as the number of current generation increases, and increases through non-improving generations. The value of *F* is always greater than 0 and smaller than 2.
5. The classifier threshold has been computed by minimizing the classifier weighted error (1 minus balanced accuracy). To do this, the response of all the training samples is ordered, and the weighted error is computed for each value between two samples. The threshold leading to the lowest weighted error is finally selected.
6. The stop criterion has been set to stop searching when the maximum number of generations, *n*_max_, (set to 500) is reached, or the maximum number of generations without improvement, *n^wi^_max_*, (set to 50) is achieved. There is an improvement when the classifier weighted error of one individual in the present generation is smaller than the smallest previous classifier weighted error.

The confusion matrix with the results obtained after training is reported in Table 4; while the sensitivity, specificity, accuracy, precision [23], and balanced accuracy [24] values are reported in Table 5.

**Figure 6:**
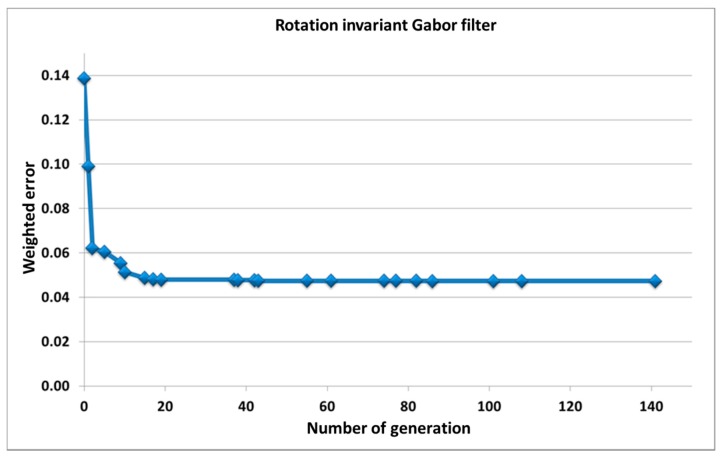
Evolution of the weighted error through the increase in the number of generations of the Gabor filter invariant to rotation.

Figure 6 shows the evolution of the weighted error through the increase in the number of generations when applying the proposed algorithm. The Gabor filter parameters obtained after applying the Differential Evolution algorithm, once the error has been stabilized (around 20th generation), are: frequency = 0.1599 (pixels^−1^), dispersion along *X* = 2.0813 (pixels), dispersion along *Y* = 17.1607 (pixels), threshold = 3.7301.

**Figure 7:**
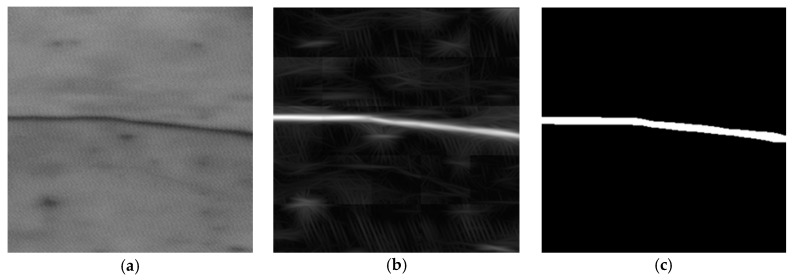

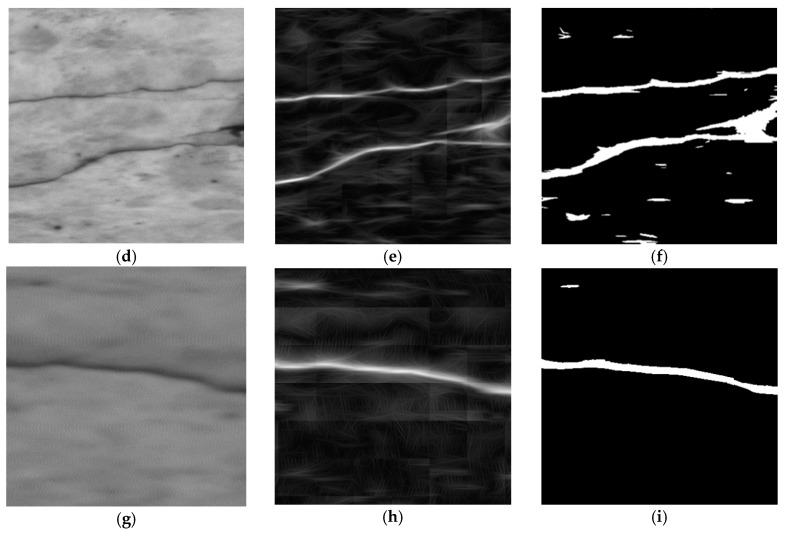
Example of application of the rotation-invariant Gabor filter. (**a**,**d**,**g**) are the normalized original images. (**b**,**e**,**h**) are the filtered images; (**c**,**f,i**) the segmented images.

The time required for the proposed algorithm to process a 1 Mbyte image is 65.6 s, which corresponds to a 15.6 kbytes/s processing speed using an Intel Pentium Core 2 Duo E8400 at 3 GHz. Figure 7 shows three examples where the described technique has been applied to 256 × 256 pixel images using the obtained parameters. The original normalized image, the filtered image, and the segmented image are shown. The values of the filtered image, in the upper row, range from 0 to 9.91, but have been rescaled to 0–255 for display purposes. Similarly, images in the middle row, ranging between 0 and 15.87, and images in the lower row, ranging between 0 and 7.89, have also been rescaled to 0–255.

A comparison of the obtained results with those obtained by other authors can be approached by taking into account the fact that, currently, there are no open datasheets available about tunnel cracks, and even the way to evaluate the results is not always the same. Some works use Gabor filters for the detection of cracks, as in [20,25]. In these works, instead of analyzing the result at pixel level, the images are divided into portions, and the detection of cracks is approached in each portion. A balanced accuracy of about 90% is reported in these works, to which the 95% obtained in this work compares favorably with [20,25]. Other works used Convolutional Neural Networks for the detection of cracks at pixel level, as can be found in [15]. The sensitivity obtained in [15] is 92.5%, while precision is 87.0%, which compares favorably to the 95.3% and 98.8%, respectively, obtained in the present paper. In the case of crack detection in the concrete surface of the tunnel, as in [8], the authors use different processing techniques and compare them to each other. The images are captured in a subway tunnel, and cracks wider than 0.3 mm and longer than 15 cm can be detected. The balanced accuracy value is not computed in this work, but the reported accuracy value, which is always equal to or greater than the balanced accuracy, ranges from 88.7% to 91.7%. Therefore, our method would compare favorably to these works.

**Figure 8:**
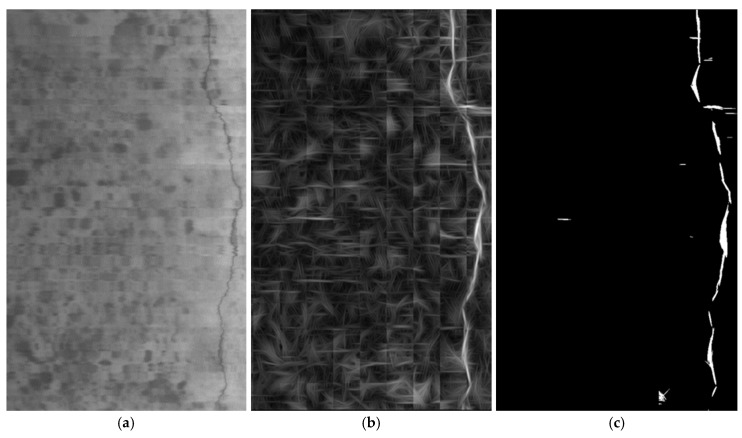
Result of applying the proposed algorithm to a large area of the tunnel. (**a**) Original image; (**b**) filtered image; (**c**) segmented image.

The proposed algorithm has also been tested with larger regions of the tunnels not used in the training process. The results obtained are satisfactory when compared to the visual inspection carried out by an expert: all cracks have been detected with a limited number of false positives. Figure 8 shows these results for a 1024 × 1714 pixel image (the maximum value is 7.09).

## 7. Conclusions

In this paper, a computer vision–based methodology for the inspection of fissures and cracks on tunnels whose surface is made of concrete is presented. A system architecture adapted to different tunnel types and a method for processing the acquired images toward detecting said cracks in the tunnel surface are proposed. 

A system prototype based on the proposed principles has been developed that uses linear sensor cameras and proper lighting, along with the software required for the system configuration and control, the data acquisition, and the display of the processing results. This prototype has been used to acquire tunnel surface images that have been used for training and testing the proposed processing method. 

This method composes a new image upon the maximum values of images filtered by a Gabor filter invariant to rotation proposed in this work, whose parameter values have been set using a modified genetic algorithm based on the Differential Evolution optimization method. The obtained results show that our method compares favorably to existing ones, including those using Gabor filters for detecting road cracks and other works focused on the detection of tunnel surface cracks.

Moreover, the eventual occurrence of shadows and humidity in real tunnels hamper those algorithms based on threshold values obtained from previous knowledge (due to the brightness variations). The algorithms based on the space-frequency analysis of the images are less sensitive to changes in image brightness, which allows better results to be obtained in these situations.

The current work is based on the analysis of the sensed visual spectrum images, but other technologies could complement the approach and widen the range of defects that could be detected. For example, the defects under analysis involve geometric anomalies that could be measured, in principle, using 3D sensors. Some advances are being made in this line, but the resolution and precision required for detecting fissures and cracks are difficult to obtain in practice, so these advances are mainly focused on obtaining the 3D profile of the tunnels [26]. On the other hand, thermographic sensors could also provide relevant information, especially in the detection of defects involving moisture, although the acquisition of such images to the required resolution and at a functional speed is still a challenge.

## Figures and Tables

**Table 1:** Summarized comparisons of related work.

References	Vision Techniques
[1,2,3,4]	Thresholding original images
[2,5,6,7,8,9,10,11]	Thresholding processed images
[6,12,13]	More elaborated threshold techniques
[14,18]	Seeds
[15]	CNN
[9,10,11,16]	Edge detectors
[17]	Genetic algorithms
[3,18]	Models
[19]	SVM
[20]	Gabor filters

**Table 2:** Crack classification on tunnels.

Crack Type	Subtype	Opening (mm)	Description
0		<0.1	Usually not taken into account
A		0.1–0.4	Do not reach the intrados steel They are of an aesthetic nature
B		0.4–2	Pass through the first reinforcement layer They are stabilized
C	C1	<0.4	Multicracked precasts Cracks without risk of fall or detachment
	C2	>0.4	Multicracked precasts Cracks with risk of detachment

**Table 3:** Number of cameras and their recommended sensor resolution for the different tunnel types.

Crack Size	Sensor Resolution (Pixels)	Electricity Tunnel			Underground Tunnel			High-Speed Train Tunnel		
		No. of Cameras		Focal Length (mm)	Nb of Cameras		Focal Length (mm)	No. of Cameras		Focal Length (mm)
		1 Pass	2 Pass		1 Pass	2 Pass		1 Pass	2 Pass	
0.1 mm	12,288	14	7	80	22	11	105	–	–	–
0.4 mm	8194	6	3	50	8	4	50	–	7	135
	6144	7	4		11	6		–	9	
1 mm	6144	–	2	28	–	3	28	–	4	50
2 mm	4096	–	–		–	2	28	–	3	28
	1024	9	5	12	–	–	–	–	–	–

**Table 4:** Confusion matrix obtained in the detection of cracks in tunnels using the Gabor filters invariant to rotation.

Confusion Matrix		Expert Classification	
		Positive	Negative
Classifier classification	Positive	TP = 19,050	FP = 3772
	Negative	FN = 950	TN = 76,228

**Table 5:** Sensitivity, specificity, accuracy, precision, and balanced accuracy in the detection of cracks in tunnels using the Gabor filters invariant to rotation.

Sensitivity	Specificity	Accuracy	Precision	Balanced Accuracy
0.9525	0.9529	0.9373	0.9876	0.9527
